# Brominated indoles from a marine mollusc inhibit inflammation in a murine model of acute lung injury

**DOI:** 10.1371/journal.pone.0186904

**Published:** 2017-10-26

**Authors:** Tarek B. Ahmad, David Rudd, Kirsten Benkendorff, Layla K. Mahdi, Kaylah-Ann Pratt, Leanne Dooley, Chuanyu Wei, Michael Kotiw

**Affiliations:** 1 Marine Ecology Research Centre, School of Environment, Science and Engineering, Southern Cross University, Lismore, NSW, Australia; 2 Centre for Health Sciences Research, University of Southern Queensland, Toowoomba, QLD, Australia; Universidade do Porto, Faculdade de Farmácia, PORTUGAL

## Abstract

New drug leads for the treatment of inflammation are urgently needed. Marine molluscs are widely used as traditional medicines for the treatment of inflammation. Here we report the positive effects of a hypobranchial gland (HBG) extract and the dominant bioactive compound 6-bromoisatin from the Muricidae mollusc *Dicathais orbita*, for reducing lipopolysaccharide (LPS) induced acute lung inflammation in a mouse model. Both 6-bromoisatin and the HBG extract suppressed the inflammatory response in mice that were pre-treated by oral gavage at 48, 24 and 1 h prior to LPS infusion. The inflammatory antagonists were tested at concentrations of 0.5 mg/g and 0.1 mg/g HBG extract and 0.1 mg/g and 0.05 mg/g 6-bromoisatin in carrier oil and all treatments reduced inflammation as indicated by a significant suppression of inflammatory markers present in bronchoalveolar lavage fluid (BALF), in comparison to LPS induced positive control mice administered the carrier oil alone (*p <* 0.0001). Tumour necrosis factor-alpha (TNFα) and interleukin-1 beta (IL-1β) levels, in addition to total protein concentration were all significantly reduced in BALF from mice treated with the extract or 6-bromoisatin. Furthermore, all treatment groups showed significant reductions in neutrophil sequestration and preservation of the lung tissue architecture compared to the positive control (*p* < 0.0001). The combined results from this study and our previous *in vitro* studies indicate that 6-bromoisatin in the HGB extracts inhibit the activation of inflammatory signalling pathway. The results from this study further confirm that the HBG extract from Muricidae molluscs and 6-bromoisatin are bioavailable and effective *in vivo*, thus have potential for development as natural therapeutic agents for inflammation.

## Introduction

Inflammation is a natural immune response to infection, but if left unchecked can lead to tissue damage and chronic disease. Acute lung inflammation (ALI) is a life-threatening syndrome that can lead to multisystem organ failure and is a significant cause of morbidity and mortality worldwide [1, 2]. A high incidence of ALI is associated with infection by Gram negative pathogenic bacteria, as these bacteria contain lipopolysaccharides (LPS) as a major component of the outer cell membrane. The Toll-Like Receptors-4 (TLR-4) found on alveolar macrophages and epithelial cells recognise LPS during lung infection and play a critical role in initiating the host’s immune response [1, 3]. This response often leads to the activation of the inflammatory nuclear transcription factor Kappa B (NFκB) pathway in the alveolar macrophages and epithelial cells, resulting in a marked increase in the production of key pro-inflammatory cytokines such as tumour necrosis factor-α (TNFα) and interleukin-1β (IL-1β) [4–6]. The production of pro-inflammatory chemokines and cytokines leads to the recruitment of neutrophils into the lung interstitium and alveolar spaces [1]. In this environment, activated neutrophils produce large amounts of free radicals and reactive oxygen species (ROS), such as nitric oxide (NO), O_2_^−^ and OH^−^, which cause pulmonary endothelial damage and damaging oxidation of lipid and protein components of cells [7]. All these events can eventually lead to impairment of gas exchange and non-cardiogenic protein-rich oedema inside the alveolar space due to the damage to the endothelial and epithelial cells [8]. Consequently, these physiological changes can eventually lead to respiratory failure and death.

There is an urgent need for anti-inflammatory agents that downregulate the release of inflammatory mediators and thus reduce the symptoms of ALI without serious side effects. Mouse models of ALI are well established for investigating acute lung injury such as respiratory distress syndrome (ARDS) in humans and for exploring potential therapeutic treatments. Airway administration of LPS leads to the development of an acute inflammatory process characterised primarily by the infiltration of neutrophils into the airspace leading to areas of haemorrhage in lung tissues, generation of pro-inflammatory cytokines and mediators, and damage to the alveolar architecture [9, 10]. The inflammatory response in the lungs peaks within the first six hours and reaches the plateau after around 12 h before the mice fully recover after approximately 72 h [10]. The mouse ALI model is well characterised and the progression of the inflammatory cascade is also well known, so novel anti-inflammatory agents can be effectively compared to established anti-inflammatory agents with known modes of action.

Many natural products are sourced from marine organisms due to their inherent broad biological and chemical diversity. Marine natural products have been shown to have an extensive array of therapeutic properties, including anticoagulant, antimicrobial, wound healing and immune modulation, antioxidant, anticancer, anti-inflammatory, antihypertensive, and other reported bioactivity [11, 12]. There is significant data demonstrating that molluscs have been used to treat inflammatory conditions in many traditional medicine regimes [13, 14]. The potential anti-inflammatory activity of molluscs has been supported by many *in vitro* and *in vivo* studies, as well as in human clinical trials. Preliminary studies on extracts from whelks in the Muricidae family of predatory gastropods indicate that these yield secondary metabolites with interesting anti-inflammatory properties [15, 16].

**Fig 1:**
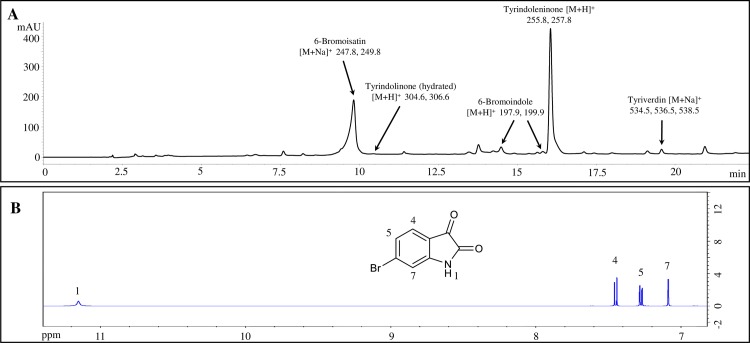
Chemical analysis of the hypobranchial gland (HBG) extract and 6-bromoisatin. A) High performance liquid chromatography (HPLC) chromatogram of the (HBG) extract from *Dicathais orbita* showing brominated indole profile. Retention times (tR) and major ions in ESI-MS indicate the presence of 6-bromoisatin (tR = 9.825 min), hydrated tyrindolinone (tR = 10.454 min), tyrindoleninone (tR = 16.056 min) and tyriverdin (tR = 19.571). B) H^1^-NMR spectra of 6-bromoisatin showing purity of the synthetic compound used in this study.

The Muricidae family of marine gastropod molluscs is well known for the production of brominated indoles [15, 17], including 6-bromoisatin (Fig 1), which has well established anti-cancer and chemopreventative properties via the induction of apoptosis [18–21]. Indoles are aromatic nitrogen-containing compounds of particular interest because of their bioactivity and pharmaceutical potential [22]. In muricid molluscs, brominated indoles are produced in a specialized biosynthetic organ called the hypobranchial gland (HGB) [23]. Both the HBG extract and 6-bromoisatin from *D*. *orbita* have anti-inflammatory activity in *in vitro* assays with evidence that they inhibit the production of inflammatory mediators and pro-inflammatory cytokines including NO, TNFα in LPS-stimulated RAW 264.7 cells, PGE2 in calcium ionophore-triggered 3T3 ccl-92 fibroblasts and significantly inhibit the translocation of NFκB into the nucleus in LPS-stimulated RAW 264.7 cells [16]. However, the anti-inflammatory activity of HBG extract and the dominant brominated indole, 6-bromoisatin, is yet to be confirmed *in vivo*. The efficacy and safety of both HBG extract and 6-bromoisatin has been validated in previous *in vivo* cancer models [20, 21, 24] and their low *in vivo* toxicity [20, 21, 25, 26] confirms the potential for development as natural drugs. Consequently, this study aims to test whether the HBG extract from the Australian Muricidae *Dicathais orbita*, along with the main bioactive constituent 6-bromoistain, can systemically ameliorate the early inflammatory response and protect lung architecture in a LPS-mediated ALI mouse model.

## Materials and methods

### Chemicals and reagents

Synthetic 6-bromoisatin was obtained from Tokyo Chemical Industry (Chuo-ku, Tokyo, Japan) with a high level of purity confirmed by ^1^H-NMR (Fig 1B). *Escherichia coli* O128:B12 LPS was obtained from Sigma (St. Louis, Missouri, United States). Pure filtered grape seed oil for use as a carrier oil was obtained from Australian Botanical Products (United States Pharmacopeia grade) (Hallam, VIC, Australia). Solvents were HPLC grade from Sigma Aldrich (St. Louis, MO, USA). Mouse TNFα ELISA kit was purchased from BD biosciences (Sparks, MD, USA). IL-1β ELISA kit was purchased from R&D Systems (Minneapolis, MN, USA). Pierce™ BCA Protein Assay was obtained from Thermo Fisher Scientific (Waltham, Massachusetts, United States). Isoflurane and Lethabarb® were purchased from Virbac Pty Ltd., (Wetherill Park, NSW, Australia).

### Preparation of mollusc extract and compound

*D*. *orbita* snails were collected from Northern New South Wales inter-tidal reefs, Australia, under Fisheries exemption permit F89/1171-6.0. Snails were kept frozen at -80°C until required. Snails were subsequently thawed and the shell carefully ruptured using a bench vice. Hypobranchial glands were excised according to the procedure described by Westley and Benkendorff [27].

Extraction of secondary metabolites from the collected HBG (40 g) was processed according to established procedures described by Edwards, Benkendorff [18]. Glands were repeatedly soaked for 2 h in solvent (chloroform: methanol, 1:1), which was replenished until a clear extract was acquired. The extract was then filtered through Whatman filter paper 1 (90 mm, Sigma-Aldrich) to remove the tissue. A chloroform/methanol partition was induced using a small amount of MilliQ water in a separation funnel. After the two phases formed, the chloroform layer was collected and kept covered in aluminium foil to protect from photolytic degradation and subsequently evaporated to dryness on a rotary evaporator (Buchi), using a vacuum pressure of 474 mbar at 40°C, then transferred to an amber vial and dried under high purity nitrogen gas. The extracts were then stored at -80°C until use.

### Liquid Chromatography-Mass Spectrometry (LC-MS) analysis

The chloroform extract of the HBG was analysed using an Agilent 1260 infinity High Performance Liquid Chromatography (HPLC) system coupled with a 6120 Quadrupole mass spectrometer (MS) according to validated procedures [28].

### Animals

All animal experiments were approved by the Animal Ethics Committee at the University of Southern Queensland (application number 15REA014). A total of 35 male and female C57 Black/6 mice were obtained from the Animal Resources Centre (ARC), Perth, Western Australia. Mice were housed in the University of Southern Queensland Animal House, maintained on an automated time cycle of 12 h light and 12 h dark and had access to rodent chow and water *ad libitum*. Mice were separated according to their gender where males were housed in a different room away from females to reduce stress. The mice were divided randomly into 6 groups (six mice per group except for the negative control group where five mice were used)

### Acute lung inflammation model

The procedure for the LPS mediated mouse model of acute lung inflammation followed the method described by Moffatt, Lever [29]. In brief, C57 Black/6 mice were randomly divided into 6 groups (n = 6 except for PBS negative control n = 5). Mice received three oral doses (administered at 48 h, 24 h and 1 h prior to the administration of LPS) of HBG extract at 0.5 mg/g or 0.1 mg/g, or 6-bromoisatin at 0.05 mg/g or 0.1 mg/g, dissolved in 100 μl analytical grade grape seed oil. Both LPS positive and negative controls received three doses of 100 μl of grape seed oil following the same timeframe. Acute lung inflammation was induced by intranasal (i.n.) administration of 1.25 mg/kg of LPS in 50 μL of sterile Phosphate buffered saline (PBS) [29–31], whilst i.n administration of 50 μL of sterile PBS was used as the negative control. Three hours post i.n. administration of LPS, mice were euthanised following isoflurane anaesthesia by intra-peritoneal (i.p.) injection of 0.2 mL Lethabarb® (pentobarbitone sodium) [31]. All handling procedures during experiments (oral gavage, intranasal administration) were performed under light anaesthesia according to USQ HP016 Rodent (rat or mouse) gaseous anaesthesia (isoflurane) to minimise stress during handling. Mice were placed inside an induction chamber supplied with 100% O_2_ (on a flow rate1.5 litres/min) and a small volume of isoflurane (4% induction and 1.5% maintenance) was supplied using an isoflurane vaporising machine (Northern Vaporisers, Skipton, UK). Respiration and response to stimulation were monitored during the procedure and the supply of isoflurane was adjusted accordingly. When returned to their allocated box, mice were monitored until complete recovery was confirmed, via active behavioural signs. Deep anaesthesia was only used prior to i.p. injection of 0.2 mL of Lethabarb® for the euthanasia process. Mice were monitored half-hourly post oral gavage for the first hour and hourly for the following 3 hrs. The monitoring was more frequent after the intranasal administration of LPS as mice were closely monitored throughout the 3 hrs post administration till euthanasia.

### Bronchoalveolar lavage fluid (BALF) collection and analysis

Following euthanasia, mice lungs were lavaged three times with 0.5 mL of ice-cold PBS as described in Fang, Gao [32]. The total cell count of the BALF was measured using an automated cell counter (BIO-RAD). The BALF was then centrifuged 1500 rpm for 10 min at 4°C. The recovered supernatant was collected and stored in -80°C freezer until use. The remaining cell pellet was resuspended in 200 μL of PBS and centrifuged in a Cytospin™ 4 (Thermo Scientific) at 700 rpm for 8 min. Cell deposits were then stained using the Diff Quick staining system (CHEMTREC®). A microscopic differential cell count was then conducted using an Olympus BX61WI microscope at 400x magnification in which 300 cells were counted on the slide (100 in three separate frames of view) and averaged to 100 cells to deduce the percentage of neutrophils (n = 6). Representative images of the cell count were captured using TUCSON camera (NIKON Japan) installed on Leica® light microscope (Leica Microsystems).

### Measurement of TNF-alpha levels in BALF

The amount of TNFα was quantified in BALF using a murine TNFα ELISA kit according to the manufacturer’s instructions. Plates were read at 405 nm wavelength using an Anthos Zenyth 200rt microplate reader (Anthos Labtech Instruments).

### Measurment of IL-1β levels in BALF

The amount of IL-1β was quantified in BALF, using mouse IL-1β ELISA kit according to the manufacturer’s instructions. Plates were read at 450 nm wavelength (Anthos Labtech Instruments) with the wavelength correction set on 540 nm.

### Total protein in BALF

Total proteins in the BALF were determined using Pierce™ BCA Protein Assay kit according to the manufacturer’s instructions.

### Lung histopathological studies

Whole lungs were harvested and fixed in 10% neutral-buffered formalin before being histologically processed in an Excelsior™ AS Tissue Processor (Thermo Scientific™), embedded in paraffin, sectioned on a microtome to 4 μm, and stained with hematoxylin and eosin (H&E). The slides were coded and assessed blind using standard histological procedures, under a light microscope for evidence of pathology, the degree of pathology subsequently compared to the negative control using a semiquantitative analysis as described by Eveillard, Soltner [33] and Klopfleisch [34]. The scores ranged from 0 (no lesion) to 4 (severe and comprehensive lesion) and were assigned according to the degree of alveolar necrosis, vascular congestion, infiltration by neutrophils, and infiltration by macrophages.

### Statistical analysis

One-way Analysis of Variance (ANOVA) followed by Dunnett’s multiple comparisons test was performed using GraphPad Prism version 6.00 for Windows (GraphPad Software, La Jolla California USA), with *p* < 0.05 considered significant. Correlation analyses between histopathological scores and bronchoalveolar lavage fluids cell counts and inflammatory markers were run in SPSS version 22 (IBM SPSS Analytics, St Leonards, NSW, Australia). The linear relationships were tested using Pearson’s correlation.

## Results

### Chemical analysis of HBG extract

LC-MS analysis of the HGB extract confirmed the presence of the brominated indoles typically found in *D*. *orbita* (Fig 1A) [17]. The extract was dominated by the presence of 6-bromoisatin (35.34%) beside tyrindoleninone (39.98%) and traces of 6-bromoindole and tyriverdin (Fig 1A). H^1^ NMR confirmed the purity of the synthetic 6-bromoisatin (Fig 1B).

### Total and differential cell counts

**Fig 2:**
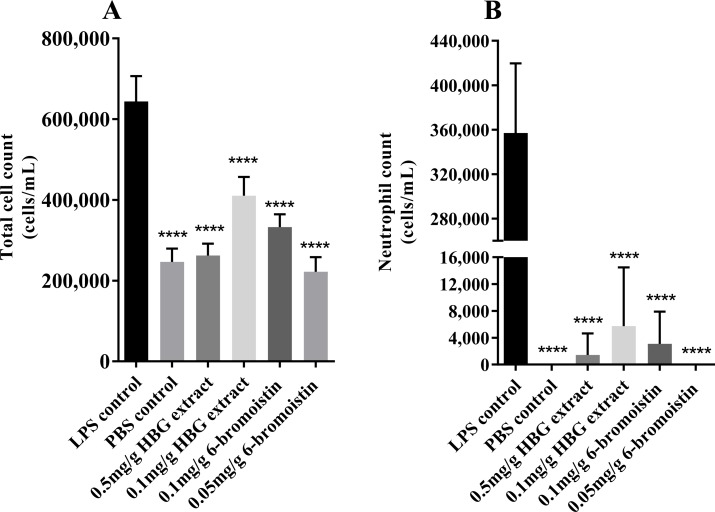
Cellularity in the bronchoalveolar lavage fluids (BALF). Cell count in the BALF from mice stimulated with LPS and treated with 6-bromoisatin or a hypobranchial gland (HBG) extract from *Dicathais orbita*. A) Total cell counts; B) Neutrophil counts from the differential staining. *** = *p <* 0.001; **** *p <* 0.0001.

The total number of cells in BALF of LPS-stimulated positive controls was 6.4 x 10^5^ cells/mL (Fig 2A). Relative to the positive control, the number of cells significantly decreased (*p* < 0.0001) in all the treatment groups: means of 2.6 x 10^5^ cells/mL in HBG extract 0.5 mg/g dose and 4.1 x10^5^ cells/mL in the 0.1 mg/g dose, and for 6-bromoisatin, means of 2.2 x 10^5^ cells/mL in the 0.05 mg/g dose and 3.3 x 10^5^ cells/mL in the 0.1 mg/g dose. All treatments except 0.1 mg/g HGB extract were not significantly different from the PBS negative control or each other (Fig 2A, *p* > 0.05).

Differential cell staining shows a high number of neutrophils in the LPS stimulated positive control compared to the negative control and all treatments (S1 Fig). Compared to the LPS positive control with neutrophil counts at a mean of 3.6 x 10^5^ cells/mL (Fig 2B), mice in all treatment groups had a significantly reduced neutrophil count (*p* < 0.0001), with a total absence of neutrophils in the 6-bromoisatin 0.05 mg/g dose regime and PBS negative control. The other treatment groups also displayed significant inhibition of neutrophil sequestration in the lungs (Fig 2B), with only 1.4 x 10^3^, 5.7 x 10^3^ and 3.1 x 10^3^ neutrophils/mL found in the BALF collected from the HBG extract at 0.5 and 0.1 mg/g, and 6-bromoisatin (0.1 mg/g) treatment groups respectively (n = 6; *p <* 0.0001). There was no significant difference between the treatment groups.

### Measurment of TNFα levels in BALF

**Fig 3:**
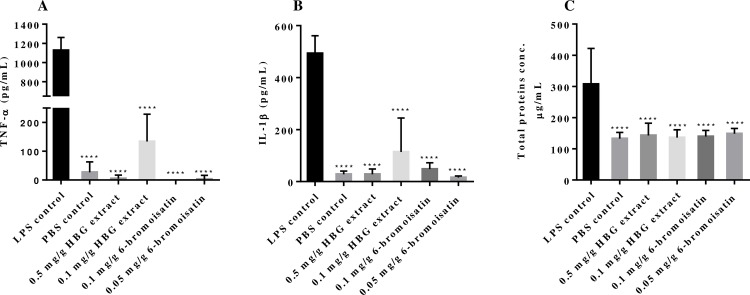
Cytokine and total protein concentration in BALF. Inhibitory effect of hypobranchial gland (HBG) extract from *Dicathais orbita* and 6-bromoisatin on the LPS-stimulated acute lung inflammation (ALI) in mice indicated by the levels of pro-inflammatory cytokines and total proteins in bronchoalveolar lavage fluids (BALF). A) Concentration of TNFα; B) IL-1β levels obtained using ELISA and; C) total protein concentration in BALF obtained by BCA assay. All BALF were collected from mice 3 h post intranasal (i.n.) administration of LPS or PBS (n = 6; **** = *p* < 0.0001). Mice received 3 doses of HBG extract (0.5 mg/g or 0.1 mg/g), 6-bromoisatin (0.1 mg/g or 0.05 mg/g) and both control groups received 100 μL of grape seed oil 48 h, 24 h and 1 h prior to i.n. administration of LPS or PBS.

The HBG extract treatment significantly decreased the level of TNFα relative to the positive control in a dose dependent manner (Fig 3A), with 5 pg/mL TNFα detected at 0.5 mg/g and 133 pg/mL TNFα in the 0.1 mg/g HBG dose, compared to the positive control yield of 1127 pg/mL (n = 6; *p*< 0.0001). No TNFα was detected following treatment with 0.1mg/g of 6-bromoisatin and only 3 pg/mL TNFα detected after pre-treatment with 0.05 mg/g 6-bromoisatin. (Fig 3A). There was no significant difference between the treatment groups.

### Measurment of IL-1β levels in BALF

The IL-1β concentration in the BALF from mice that received 0.5 mg/g HBG extract was equal to the level detected in the PBS negative control at 28 pg/mL and significantly lower than the 493 pg/mL in the LPS positive control (*p* < 0.0001, Fig 3B). The lower dose (0.1 mg/g) of HBG extract also reduced the levels of IL-1β significantly (114 pg/mL) compared to the LPS positive control. Treatment with 0.05 mg/g 6-bromoisatin reduced the concentration of IL-1β to 16 pg/mL (Fig 3B). All treatments were significantly lower than the LPS positive control but not from each other.

### LPS-induced protein concentration in BALF

After i.n. LPS-administration, protein concentration in BALF from mice significantly increased as a marker of leakage, and was 307 μg/mL after 3 h compared with the PBS control, which was only at 133 μg/mL (Fig 3C). The BALF protein concentration was reduced in LPS-stimulated mice treated with the *D*. *orbita* HBG extract and 6-bromoisatin, reaching only 139 μg/mL in mice receiving 0.1 mg/g 6-bromoisatin and 136 μg/mL in mice receiving 0.1 mg/g HBG extract. The difference between all treatment groups versus the LPS positive control was significant (*p* < 0.0001), but not different from the baseline protein concentrations in the BALF of negative controls (Fig 3C). There was a significant dose effect for 6-bromoisatin with slightly higher protein levels at 0.05 compared to 0.1 mg/mL (p = 0.018).

### Histopathology of the lungs

**Fig 4:**
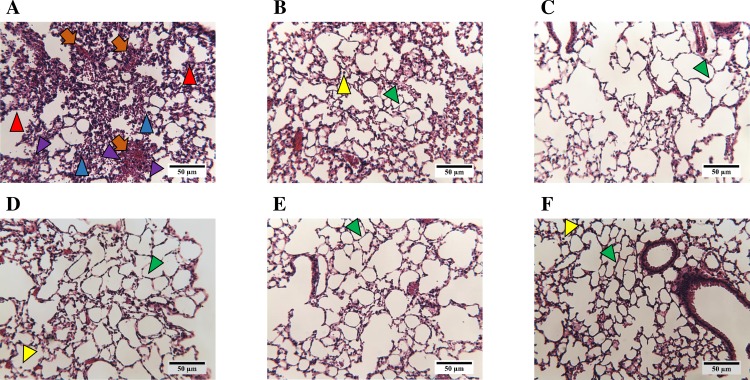
Muricid hypobranchial gland extract and 6-bromoisatin treatment protect lung tissues from the adverse effect of LPS. H&E stained lung sections demonstrating the anti-inflammatory effects of hypobranchial gland (HBG) extract from *Dicathais orbita* and 6-bromoisatin on LPS-induced acute lung inflammation (ALI). Lung tissue from A) LPS stimulated positive control showing areas of severe haemorrhage (orange arrows), infiltration of macrophages (blue arrow heads) and neutrophils (purple arrow heads), thickened alveolar walls (red arrow heads). The unstimulated negative control mice that received 50 μL PBS intranasally (i.n.) (B), 0.5 mg/g HBG extract treated mice (C), 0.1 mg/g HBG extract treated mice with undamaged alveolar space and parenchymal architecture (D), mice treated with 0.1 mg/g of 6-bromoisatin (E), and mice treated with 0.05 mg/g 6-bromoisatin (F) all showing preserved alveolar space and parenchymal architecture and all showed thin alveolar wall (green arrow heads) and lack of signs of macrophage and neutrophil infiltrations, except for occasional alveolar macrophages (yellow arrow heads). Images are representative of 3 different sections on each slide out of 6 slides per group magnified X 200 and scale bars set to 50 μm.

Examination of histological sections of the lungs from the LPS positive control group revealed areas of severe haemorrhages (indicated by orange arrows), damage to the lung architecture, increased cellularity in the air spaces, and clear evidence of both macrophage (blue arrow heads) and neutrophil (purple arrow heads) infiltration, with a large number of macrophages and neutrophils found in the alveolar spaces (Fig 4A and S2 Fig). Lung histopathology examinations also revealed damage to and thickening of alveolar walls (red arrow heads) in tissue sections from the LPS positive control mice (Table 1 and Fig 4A). In contrast, damage to lung tissues was greatly reduced in all HBG extract and 6-bromoisatin treated mice. The treated mice showed lung architecture similar to the negative control group, displaying normal lung architecture with no evidence of haemorrhage or neutrophil sequestration into the airspaces, apart from occasional alveolar macrophages (yellow arrow heads) and relatively thin alveolar walls (green arrow heads) (Fig 4B–4F, S2 Fig and Table 1). Semi-quantitative scoring of the microscopic histopathological examination confirmed that treatment with all doses of HBG extract and 6-bromoisatin significantly minimised all the indicators of acute inflammatory damage to the lungs, including macrophage and neutrophil infiltration into the airspace, as well as vascular congestion and alveolar destruction, compared to the LPS positive control group (*p* < 0.0001) (Table 1). There was an inverse dose response for 6-bromoisatin with significantly higher macrophages (p = 0.006) at 0.1mg/g compared to the lower dose of 0.05mg/g.

**Table 1:** Histopathological scores for lungs tissues.

Treatment Group	Macrophage Infiltration	Neutrophil Infiltration	Vascular Congestion	Alveolar Destruction	Total Score
**LPS control**	3.8 ± 0.2	3.7 ± 0.2	3.2 ± 0.4	2.9 ± 0.4	13.5 ± 1.2
**PBS control**	1.4 ± 0.2****	0 ± 0****	0.4 ± 0.2****	0.6 ± 0.2****	2.4 ± 0.7****
**0.5 mg/g HBG**	2 ± 0.4****	0.2 ± 0.2****	1 ± 0.3****	1.5 ± 0.2**	4.7 ± 0.6****
**0.1 mg/g HBG**	2.8 ± 0.2****	0.5 ± 0.3*	0.8 ± 0.3****	0.7 ± 0.2****	4.8 ± 0.8****
**0.1 mg/g 6-bromoisatin**	2.6 ± 0.2****	0.3 ±0.2**	0.7 ± 0.2****	0.7 ± 0.2****	4.3 ± 0.7****
**0.05 mg/g 6-bromoisatin**	1.5 ± 0.2****	0 ± 0****	0.3 ± 0.2****	0.5 ± 0.2****	2.3 ± 0.3****

Histopathological scores for lungs of mice after LPS stimulation (PBS control received 50 μL of PBS intranasally (i.n.) instead of LPS) and oral gavage with extracts from the hypobranchial glands of *Dicathais orbita* or the dominant secondary metabolite 6-bromoisatin. Data are means ± standard error of the means, n = 6 for all groups except for PBS control n = 5.

* = *p* < 0.05

** = *p* < 0.01

**** = *p* < 0.0001.

### Correlation between histopathology and inflammatory markers

**Fig 5:**
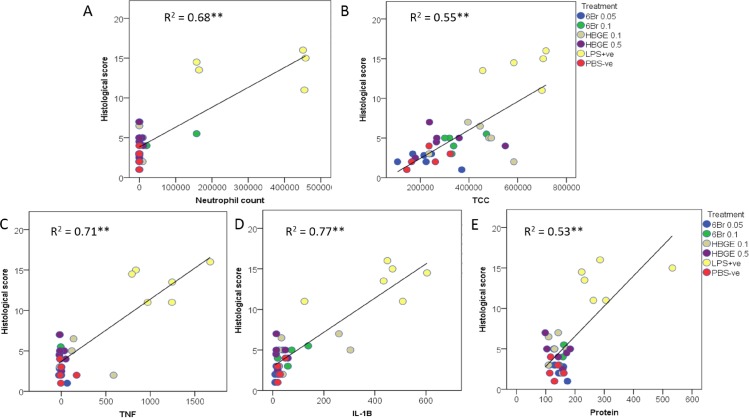
Correlations between the histological score and inflammatory markers in the lungs of mice. The relationship between the overall histopathological score and parameters in the bronchoalveolar lavage fluids: A) Neutrophil count; B), total cell count (TCC); C) Tumour necrosis factor (TNF) alpha; D) interleukin I-1B; and E) protein. The linear relationship and R^2^ values are determined from all samples pooled across all mice in the LPS-stimulated acute lung inflammation model: PBS–ve negative control; LPS +ve lipopolysaccharide stimulated positive control; HBGE Hypobranchial gland extract at 0.1 and 0.5 mg/g and; 6Br 6 bromoisatin at 0.1 and 0.05 mg/g. ** significant relationship at p < 0.01.

There were significant positive correlations between histopathological scores and inflammatory markers in the bronchoalveolar lavage fluids (p < 0.001, Fig 5 and S3–S8 Figs). Each of the histopathological markers was significantly correlated to the total semi-quantitative score (S3 Fig). The total score is significantly correlated to the cellularity and cytokines in the BALF (Fig 5) and demonstrates that the correlations are primarily driven by the higher scores and BALF values in the LPS positive control, compared to the negative control and all treatment groups. The correlations tend to be stronger for neutrophils than total cell count for all histopathological scores (Fig 5 and S4–S7 Figs). The correlations are also stronger for the cytokines TNFα and IL-1B than total protein (S4–S7 Figs) and greater than 70% of the variation in the total histological score is explained by these individual cytokines (Fig 5). Within the BALF, cell numbers were significantly correlated to the cytokines and total protein, with the strongest relationships found between neutrophils and TNFα (S8 Fig).

## Discussion

The present study has demonstrated the anti-inflammatory properties of orally administered HBG extract from the marine mollusc *D*. *orbita* and the major constituent 6-bromoisatin in a LPS mediated mouse model of acute lung inflammation. These findings support previous evidence of *in vitro* anti-inflammatory activity [16] and suggest that HBG extract from muricid molluscs and associated brominated indoles should be explored further as orally active therapeutic alternatives for the treatment of lung inflammation. ALI is a life threatening condition associated with high levels of morbidity and mortality. Despite the significance of these diseases, there has been little progress in the development of alternative treatment regimens to the conventional use of steroids, current NSAID supplemented with antibiotics and assisted ventilation [6, 35].

Isatins are naturally occurring molecules and are well known for their synthetic versatility and diverse pharmacological properties [36, 37]. 6-Bromoisatin is a brominated form of isatin, which has been shown to have *in vitro* anti-inflammatory activity in our previous studies [16]. Modes of action for simple isatin derivatives appear to be associated with inhibiting the transcription of iNOS and COX-2 enzymes [38] and the translocation of NFκB, thus suppressing NO and TNFα [16] in mouse macrophage cell lines. Halogenated isatins have been shown to have greater biological activity than non-halogenated forms, as demonstrated by structure-activity relationship studies [16, 39]. As far as we are aware, this is the first study to establish that bromoisatin is active in an animal model for acute inflammation.

Interestingly a tendency towards a reverse dose effect was observed for 6-bromoisatin in the histopathological scores, with a significant difference between doses for macrophage infiltration. Similar reverse dose effects have been observed with synthetic 6-bromoisatin in a 14 day mouse model for colon cancer, specifically for apoptosis [20] and for haematological white blood cell and neutrophil counts using 6 bromoisatin semi-purified from *Dicathais orbita* extracts [21]. It is uncertain why this occurs, but it is most likely the result of *in vivo* metabolism and degradative reactions that occur at high versus low doses. 6-Bromoisatin can form dimers *in vivo* [40] and higher concentrations would increase the opportunity for dimers to form. As reported in our previous *in vitro* studies, brominated indole dimers have lower anti-inflammatory activity than the monomers [16].

**Fig 6:**
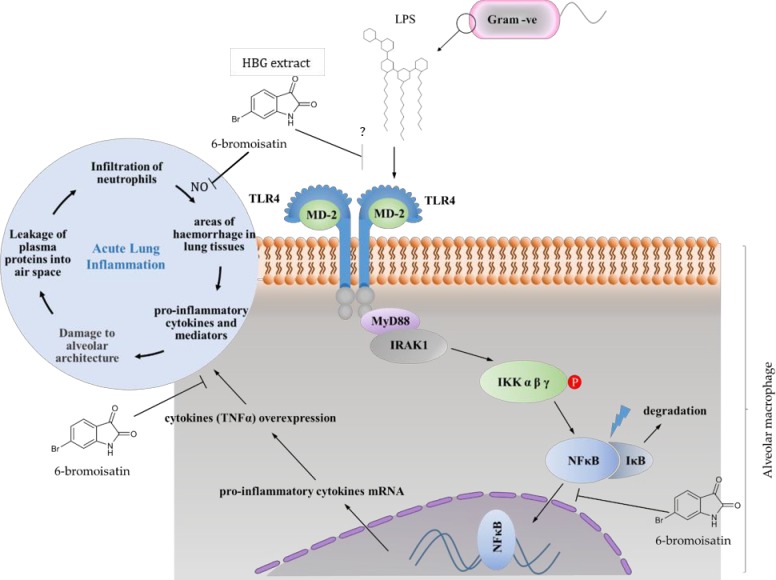
Proposed model of anti-inflammatory signalling pathway inhibition. 6-Bromoisatin in *D*. *orbita* hypobranchial gland (HGB) extracts prevent acute lung damage caused by inflammatory neutrophils by reducing the synthesis of pro-inflammatory cytokines. This may occur due to blocking the LPS-induced NFκB translocation into the nucleus and activation of macrophages and direct inhibition of inflammatory mediators, such as TNFα and nitric oxide (NO), as has been previously demonstrated *in vitro* for 6-bromoisatin [16]. Alternatively, it is possible that the HGB extracts and associated compounds also have an upstream effect by modulating the interaction of LPS with plasma membrane receptors. This figure was developed from known inflammatory pathways in lung macrophages [3].

All of the semi-quantitative histological parameters and inflammatory markers from the bronchoalveolar lavage fluids were significantly correlated in this study and provide multiple lines of evidence supporting the inhibition of LPS-stimulated inflammation and associated damage to the lungs by 6-bromoisatin and the natural HBG extract. According to available results from this study and our previous *in vitro* study [16], 6-bromoisatin appears to inhibit the overproduction of pro-inflammatory mediators and cytokines by inhibiting the translocation of NFκB (Fig 6). Translocation of NFκB to the nucleus regulates the expression of a variety of transcription factors and co-factors, that lead to the expression of pro-inflammatory enzymes including COX-2 and iNOS, which are responsible for stimulating further signalling molecules (Fig 6), including adhesion molecules, pro-inflammatory cytokines (IL-1β, IL-6, and TNFα) and chemokines [41–43]. It is well known that unresolved inflammatory responses can cause serious damage to the affected tissues, as well as the neighbouring tissues. Hence, agents that inhibit NFκB translocation and /or the whole TLR4 signalling pathway could be of a significant value as anti-inflammatory agents and may prevent the tissue damage caused during ALI. Nevertheless, based on the current *in vivo* model, we can’t rule out potential upstream effects, such as interference with LPS binding to receptors on the surface of cells (Fig 6). Further studies are required to elucidate the specific mode/s of action and investigate the pharmacokinetics of 6-bromisatin using different treatment regimes.

The potential for therapeutic use of *D*. *orbita* HBG chloroform extract and 6-bromoisatin as anti-inflammatory agents also relies on low *in vivo* toxicity. In previous rodent models, there was no morbidity, ill health or gastro-intestinal damage apparent after 2–14 weeks of daily oral gavage with the muricid extract or pure 6-bromoisatin [20, 21, 25, 26, 40]. As gastro-intestinal damage is the most significant side effect of currently used treatments (NSAIDs) [44], 6-bromoisatin provides a lead for the development of safer anti-inflammatory drugs. In addition to the anti-inflammatory activity reported here, *D*. *orbita* chloroform extracts and brominated indoles have been shown to harbour antibacterial properties against a range of Gram positive and negative bacteria [45]. Furthermore, 6-bromoisatin has anti-proliferation and apoptotic properties and effectively prevents early stage colorectal cancer formation in rodents [20, 21]. Likewise, HBG extracts also induce apoptotic effects in DNA damaged cells in colorectal cancer rodent models [21, 24]. However, some of the other brominated indoles in the HGB extracts appear to cause idiosyncratic liver damage [21, 25], suggesting that there are benefits associated with using purified 6-bromoisatin. Furthermore, polar extracts from Muricidae hypobranchial glands contain choline esters which can be associated with significant toxicity [15, 46]. However, Rudd and Benkendorff [47] have published a method for supercritical fluid extraction of *D*. *orbita* HGBs that concentrates the anti-inflammatory brominated indoles without the toxic choline esters. This safe extraction method is suitable for future nutraceutical development and will facilitate quality control of the natural product.

Rodent models of ALI are well established and have been extensively used to explore the complex pathobiology of this syndrome. Activated neutrophils in the airways are the major risk that leads to weakened lung pathology [31, 48]. In this study, oral treatment with HBG extract and 6-bromoisatin showed significant suppression of neutrophil sequestration, although the full mode of action of these inflammatory antagonists needs further exploration. Furthermore, IL-1β is considered one of the most potent pro-inflammatory cytokines [49]. This cytokine has been shown to have more potent activity than TNFα in inducing fever, in addition to its many other effects on endothelial cells, such as promoting coagulation and thrombosis, promoting infection-related and injury-related inflammation, pain hypersensitivity [50], and induction of collagenase production, which contributes to the induction of a range of inflammatory diseases [49]. IL-1β is also known to play a significant role in the pathogenesis of type-1 diabetes [51], acute neurodegeneration, stroke, tumour angiogenesis and invasiveness [52], and destructive joint and bone diseases [49]. Thus, reduction of IL-1β levels by the HBG extract could contribute to the broad range of traditional medicinal applications previously reported for Muricidae molluscs [15], including preparations that have been used to treat asthma, cough and reduction of respiratory phlegm [53–57]. Both the HBG extract and 6-bromoisatin also significantly inhibited the leakage of proteins into the air spaces in the lungs, as supported by the normal protein concentrations in the collected BALF. These results were supported by the histopathological findings where clear healthy lung tissues were observed for mice pre-treated with HBG extract and 6-bromoisatin.

In conclusion, the anti-inflammatory activity of 6-bromoisatin and the HBG extract from *D*. *orbita* in a mouse model for ALI supports previous *in vitro* observations of the anti-inflammatory capability of these agents [16]. Although a number of brominated indoles have been characterised in the HBG extract from *D*. *orbita*, 6-bromoisatin is considered safer [21] and easier to synthesise [39]. Several studies support the safety of oral consumption of this compound and the extracts in rodents, which suggests the potential for development as nutraceutical anti-inflammatory preparations. Further studies to elaborate the activity of HBG extract from *D*. *orbita* and 6-bromoisatin in particular seems warranted.

## Supporting information

S1 Fig Composition and the morphology of the cellularity in BALF. A) LPS stimulated untreated mice; B) unstimulated untreated mice; C) mice stimulated and treated with 0.5 mg/g HBG extract; D) mice treated with 0.1 mg/g HBG extract; E) mice treated with 0.1 mg/g 6-bromoisatin; F) mice treated with 0.05 mg/g 6-bromoisatin. Alveolar macrophages (blue arrows) are the dominant cells with unsegmented nucleus and the neutrophils (purple arrows) are plenty in the LPS stimulated positive control, while very scanty or absent in the other groups. Images are representative of three different fields from a total of 6 mice per group except for the PBS negative group n = 5. Scale bars set to 50 μm.
(TIF)

S2 Fig Microscopic examination of the H&E stained lung sections. A) LPS positive control showing the areas severe haemorrhage as indicated by large amount of red blood cells in the air space (brown arrows), thickened and damaged alveolar walls (yellow arrows), infiltration of alveolar macrophages (blue arrows) and neutrophils (purple arrows) into the air space; B) negative control, which received 50 uL of PBS intranasally; C) 0.5 mg/g HBG extract treated mice; D) 0.1 mg/g HBG extract treated mice with undamaged alveolar space and parenchymal architecture, E) mice treated with 0.1 mg/g of 6-bromoisatin, and; F) mice treated with 0.05 mg/g 6-bromoisatin. All HBG extract and 6-bromoisatin treated mice show preserved alveolar space and parenchymal architecture with thin alveolar walls (green arrow heads) and lack of signs of haemorrhage or macrophage and neutrophil infiltration. Images are representative of 3 different sections from six mice per group magnified 400X and scale bars set to 20 μm.
(TIF)

S3 Fig Correlations between histological parameters in the lungs of mice. The relationship between the overall histopathological score and A) neutrophil infiltration; B), macrophage infiltration; C) vascular congestion; D) alveolar destruction. The linear relationship and R2 values are determined from all samples pooled across all mice in the LPS-stimulated acute lung inflammation model: PBS–ve negative control; LPS +ve lipopolysaccharide stimulated positive control; HBGE Hypobranchial gland extract at 0.1 and 0.5 mg/g and; 6Br 6 bromoisatin at 0.1 and 0.05 mg/g. ** significant relationship at p < 0.01.
(TIF)

S4 Fig Correlations between neutrophil infiltration and inflammatory markers in the lungs of mice. The relationship between the histopathological score for neutrophils and parameters in the bronchoalveolar lavage fluids: A) Neutrophil count; B), total cell count (TCC); C) Tumor necrosis factor (TNF) alpha; D) interleukin I-1B; and E) protein. The linear relationship and R2 values are determined from all samples pooled across all mice in the LPS-stimulated acute lung inflammation model: PBS–ve negative control; LPS +ve lipopolysaccharide stimulated positive control; HBGE Hypobranchial gland extract at 0.1 and 0.5 mg/g and; 6Br 6 bromoisatin at 0.1 and 0.05 mg/g. ** significant relationship at p < 0.01.
(TIF)

S5 Fig Correlations between macrophages and inflammatory markers in the lungs of mice. The relationship between the histopathological score for macrophages infiltration and parameters in the bronchoalveolar lavage fluids: A) Neutrophil count; B), total cell count (TCC); C) Tumor necrosis factor (TNF) alpha; D) interleukin I-1B; and E) protein. The linear relationship and R2 values are determined from all samples pooled across all mice in the LPS-stimulated acute lung inflammation model: PBS–ve negative control; LPS +ve lipopolysaccharide stimulated positive control; HBGE Hypobranchial gland extract at 0.1 and 0.5 mg/g and; 6Br 6 bromoisatin at 0.1 and 0.05 mg/g. ** significant relationship at p < 0.01.
(TIF)

S6 Fig Correlations between alveolar destruction and inflammatory markers in the lungs of mice. The relationship between the histopathological score for alveolar destruction and parameters in the bronchoalveolar lavage fluids: A) Neutrophil count; B), total cell count (TCC); C) Tumor necrosis factor (TNF) alpha; D) interleukin I-1B; and E) protein. The linear relationship and R2 values are determined from all samples pooled across all mice in the LPS-stimulated acute lung inflammation model: PBS–ve negative control; LPS +ve lipopolysaccharide stimulated positive control; HBGE Hypobranchial gland extract at 0.1 and 0.5 mg/g and; 6Br 6 bromoisatin at 0.1 and 0.05 mg/g. ** significant relationship at p < 0.01.
(TIF)

S7 Fig Correlations between vascular congestion and inflammatory markers in the lungs of mice. The relationship between the histopathological score for vascular congestion and parameters in the bronchoalveolar lavage fluids: A) Neutrophil count; B), total cell count (TCC); C) Tumor necrosis factor (TNF) alpha; D) interleukin I-1B; and E) protein. The linear relationship and R2 values are determined from all samples pooled across all mice in the LPS-stimulated acute lung inflammation model: PBS–ve negative control; LPS +ve lipopolysaccharide stimulated positive control; HBGE Hypobranchial gland extract at 0.1 and 0.5 mg/g and; 6Br 6 bromoisatin at 0.1 and 0.05 mg/g. ** significant relationship at p < 0.01.
(TIF)

S8 Fig Correlations between cell counts and inflammatory markers in the bronchoalveolar lavage fluids of mice. The relationship between total cell count (top panels) or neutrophils (bottom panels) and: A & D) Tumor necrosis factor alpha; B & E) interleukin 1B; and C & F) protein. The linear relationship and R2 values are determined from all samples pooled across all mice in the LPS-stimulated acute lung inflammation model: PBS–ve negative control; LPS +ve lipopolysaccharide stimulated positive control; HBGE Hypobranchial gland extract at 0.1 and 0.5 mg/g and; 6Br 6 bromoisatin at 0.1 and 0.05 mg/g. ** significant relationship at p < 0.01.
(TIF)

S1 File NC3Rs Animal Research: Reporting In Vivo Experiments (ARRIVE) Checklist. (PDF)
