# Supplementary figures and images for: Brominated indoles from a marine mollusc inhibit inflammation in a murine model of acute lung injury
Source: PLoS One. 2017 Oct 26;12(10):e0186904. doi: 10.1371/journal.pone.0186904 (PMC5658094; doi:10.1371/journal.pone.0186904)

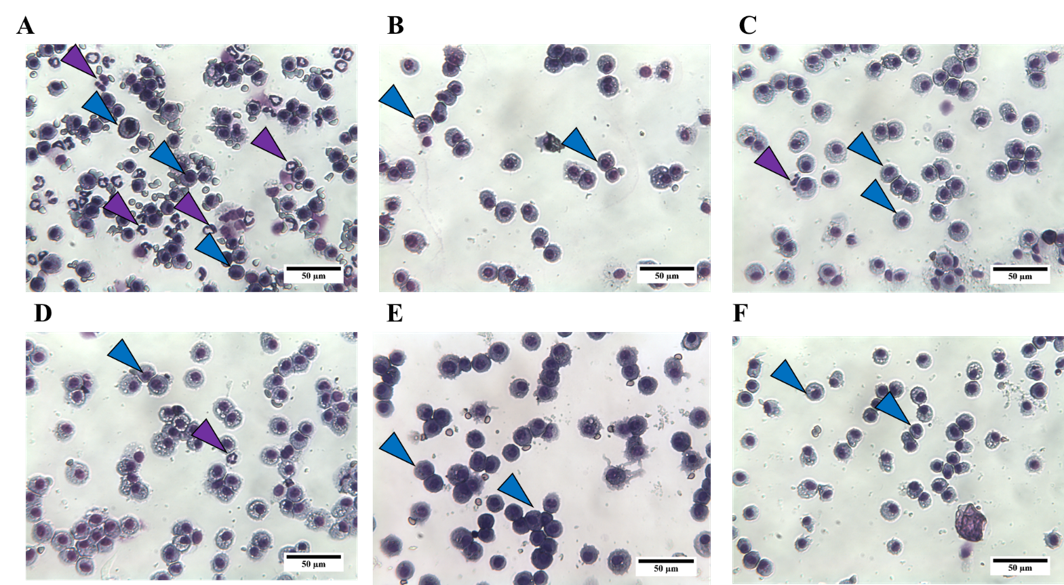

Supplement: S1 Fig — A) LPS stimulated untreated mice; B) unstimulated untreated mice; C) mice stimulated and treated with 0.5 mg/g HBG extract; D) mice treated with 0.1 mg/g HBG extract; E) mice treated with 0.1 mg/g 6-bromoisatin; F) mice treated with 0.05 mg/g 6-bromoisatin. Alveolar macrophages (blue arrows) are the dominant cells with unsegmented nucleus and the neutrophils (purple arrows) are plenty in the LPS stimulated positive control, while very scanty or absent in the other groups. Images are representative of three different fields from a total of 6 mice per group except for the PBS negative group n = 5. Scale bars set to 50 μm. (TIF) [file pone.0186904.s001.tif]

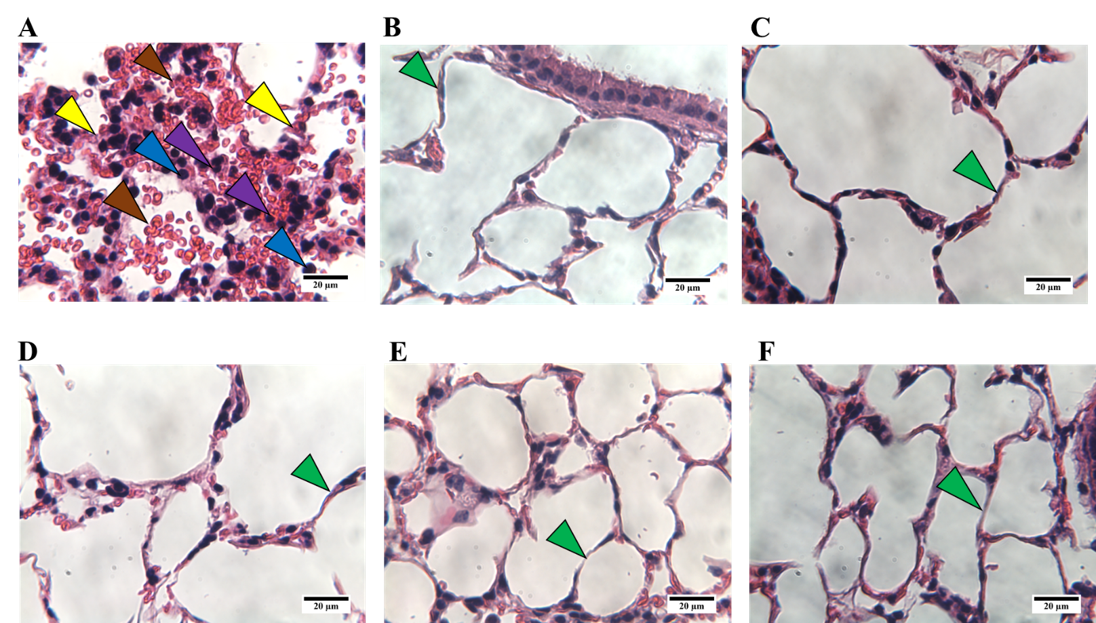

Supplement: S2 Fig — A) LPS positive control showing the areas severe haemorrhage as indicated by large amount of red blood cells in the air space (brown arrows), thickened and damaged alveolar walls (yellow arrows), infiltration of alveolar macrophages (blue arrows) and neutrophils (purple arrows) into the air space; B) negative control, which received 50 uL of PBS intranasally; C) 0.5 mg/g HBG extract treated mice; D) 0.1 mg/g HBG extract treated mice with undamaged alveolar space and parenchymal architecture, E) mice treated with 0.1 mg/g of 6-bromoisatin, and; F) mice treated with 0.05 mg/g 6-bromoisatin. All HBG extract and 6-bromoisatin treated mice show preserved alveolar space and parenchymal architecture with thin alveolar walls (green arrow heads) and lack of signs of haemorrhage or macrophage and neutrophil infiltration. Images are representative of 3 different sections from six mice per group magnified 400X and scale bars set to 20 μm. (TIF) [file pone.0186904.s002.tif]

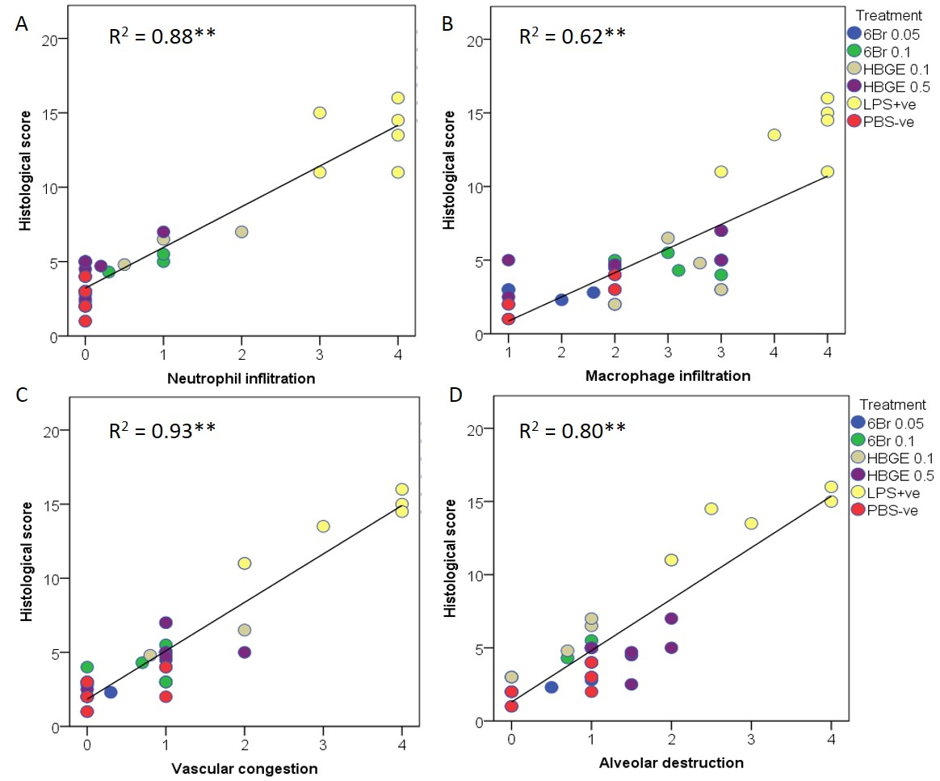

Supplement: S3 Fig — The relationship between the overall histopathological score and A) neutrophil infiltration; B), macrophage infiltration; C) vascular congestion; D) alveolar destruction. The linear relationship and R2 values are determined from all samples pooled across all mice in the LPS-stimulated acute lung inflammation model: PBS–ve negative control; LPS +ve lipopolysaccharide stimulated positive control; HBGE Hypobranchial gland extract at 0.1 and 0.5 mg/g and; 6Br 6 bromoisatin at 0.1 and 0.05 mg/g. ** significant relationship at p < 0.01. (TIF) [file pone.0186904.s003.tif]

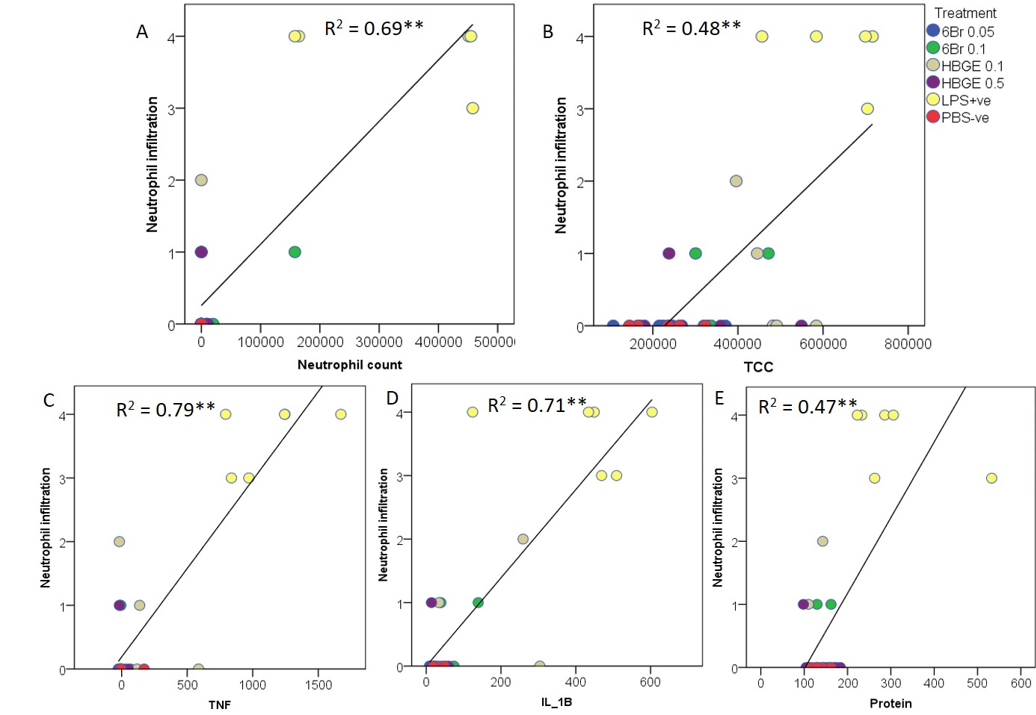

Supplement: S4 Fig — The relationship between the histopathological score for neutrophils and parameters in the bronchoalveolar lavage fluids: A) Neutrophil count; B), total cell count (TCC); C) Tumor necrosis factor (TNF) alpha; D) interleukin I-1B; and E) protein. The linear relationship and R2 values are determined from all samples pooled across all mice in the LPS-stimulated acute lung inflammation model: PBS–ve negative control; LPS +ve lipopolysaccharide stimulated positive control; HBGE Hypobranchial gland extract at 0.1 and 0.5 mg/g and; 6Br 6 bromoisatin at 0.1 and 0.05 mg/g. ** significant relationship at p < 0.01. (TIF) [file pone.0186904.s004.tif]

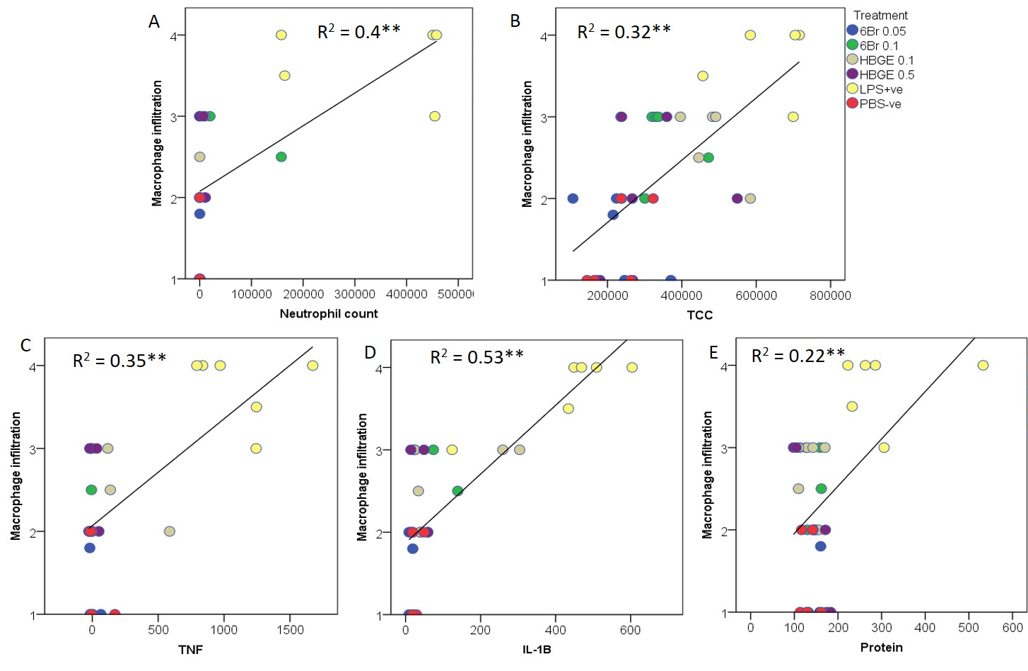

Supplement: S5 Fig — The relationship between the histopathological score for macrophages infiltration and parameters in the bronchoalveolar lavage fluids: A) Neutrophil count; B), total cell count (TCC); C) Tumor necrosis factor (TNF) alpha; D) interleukin I-1B; and E) protein. The linear relationship and R2 values are determined from all samples pooled across all mice in the LPS-stimulated acute lung inflammation model: PBS–ve negative control; LPS +ve lipopolysaccharide stimulated positive control; HBGE Hypobranchial gland extract at 0.1 and 0.5 mg/g and; 6Br 6 bromoisatin at 0.1 and 0.05 mg/g. ** significant relationship at p < 0.01. (TIF) [file pone.0186904.s005.tif]

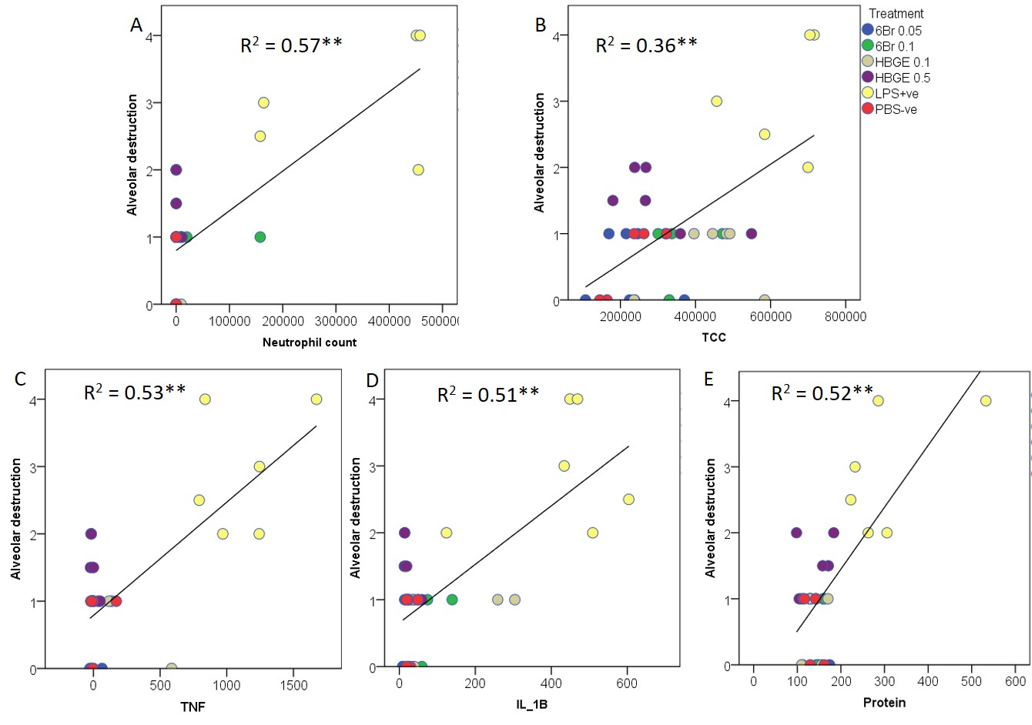

Supplement: S6 Fig — The relationship between the histopathological score for alveolar destruction and parameters in the bronchoalveolar lavage fluids: A) Neutrophil count; B), total cell count (TCC); C) Tumor necrosis factor (TNF) alpha; D) interleukin I-1B; and E) protein. The linear relationship and R2 values are determined from all samples pooled across all mice in the LPS-stimulated acute lung inflammation model: PBS–ve negative control; LPS +ve lipopolysaccharide stimulated positive control; HBGE Hypobranchial gland extract at 0.1 and 0.5 mg/g and; 6Br 6 bromoisatin at 0.1 and 0.05 mg/g. ** significant relationship at p < 0.01. (TIF) [file pone.0186904.s006.tif]

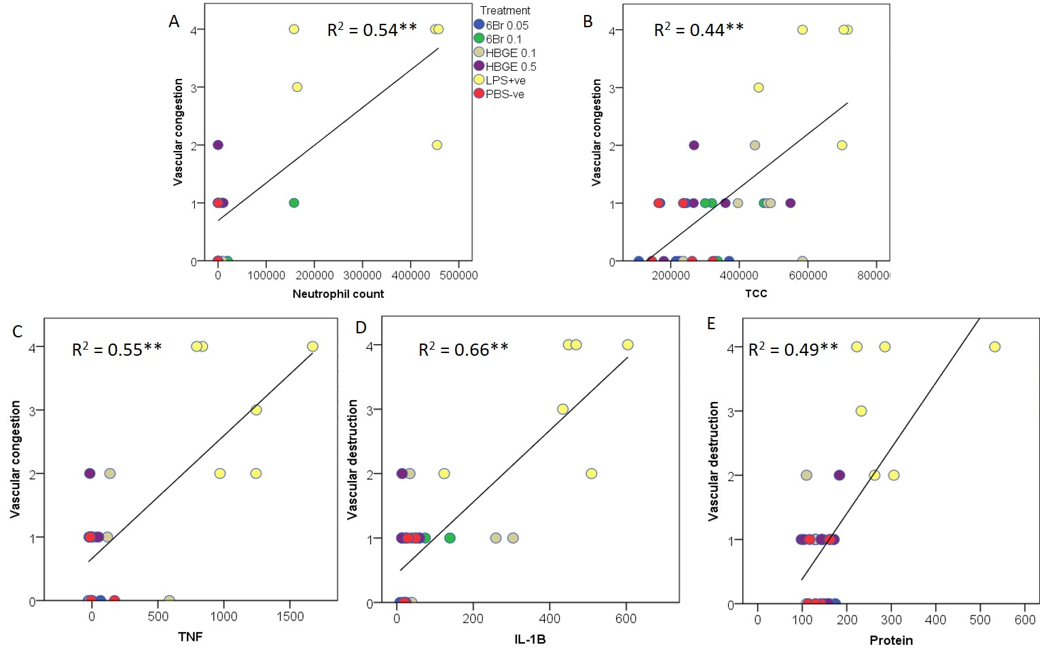

Supplement: S7 Fig — The relationship between the histopathological score for vascular congestion and parameters in the bronchoalveolar lavage fluids: A) Neutrophil count; B), total cell count (TCC); C) Tumor necrosis factor (TNF) alpha; D) interleukin I-1B; and E) protein. The linear relationship and R2 values are determined from all samples pooled across all mice in the LPS-stimulated acute lung inflammation model: PBS–ve negative control; LPS +ve lipopolysaccharide stimulated positive control; HBGE Hypobranchial gland extract at 0.1 and 0.5 mg/g and; 6Br 6 bromoisatin at 0.1 and 0.05 mg/g. ** significant relationship at p < 0.01. (TIF) [file pone.0186904.s007.tif]

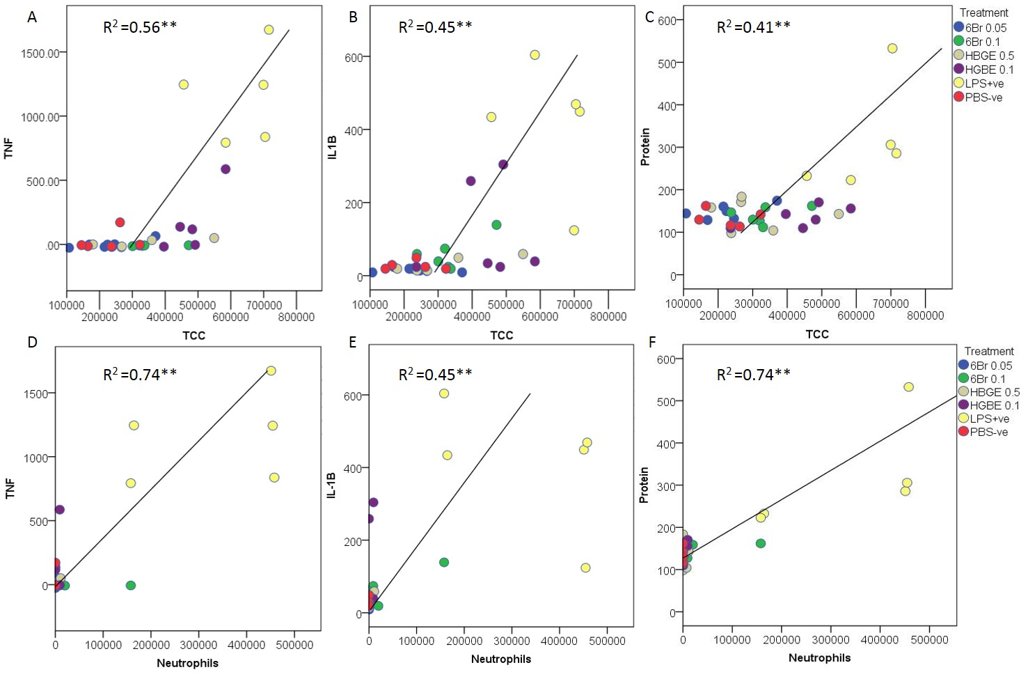

Supplement: S8 Fig — The relationship between total cell count (top panels) or neutrophils (bottom panels) and: A & D) Tumor necrosis factor alpha; B & E) interleukin 1B; and C & F) protein. The linear relationship and R2 values are determined from all samples pooled across all mice in the LPS-stimulated acute lung inflammation model: PBS–ve negative control; LPS +ve lipopolysaccharide stimulated positive control; HBGE Hypobranchial gland extract at 0.1 and 0.5 mg/g and; 6Br 6 bromoisatin at 0.1 and 0.05 mg/g. ** significant relationship at p < 0.01. (TIF) [file pone.0186904.s008.tif]
